# Metabolic profiling of human melanoma cell lines with high and low metastatic capacity by ^1^H-NMR spectroscopy

**DOI:** 10.1371/journal.pone.0352639

**Published:** 2026-07-01

**Authors:** Nima Rezvani Kakhki, Zita Hegedűs, József Tóvári, Arash Mirzahosseini, Béla Noszál, Márta Kraszni

**Affiliations:** 1 Department of Pharmaceutical Chemistry, Semmelweis University, Budapest, Hungary; 2 Center for Pharmacology and Drug Research & Development, Semmelweis University, Budapest, Hungary; 3 National Korányi Institute of Pulmonology, Budapest, Hungary; 4 Department of Experimental Pharmacology and the National Tumor Biology Laboratory, National Institute of Oncology, Budapest, Hungary; University of New Hampshire, UNITED STATES OF AMERICA

## Abstract

**Background:**

Melanoma is one of the most aggressive forms of skin cancer due to its high metastatic potential and mortality rate. Although understanding of metabolic reprogramming in melanoma has advanced, the connection between metabolic alterations and metastatic capacity remains incomplete.

**Aim:**

This study aimed to characterize the metabolic profiles of human melanoma cell lines with high (HT168-M1) and low (WM983B) metastatic potential, and to compare them with each other and also with the metabolic profile of normal human fibroblasts (MRC-5), in order to identify key metabolites and metabolic pathways associated with metastatic behavior.

**Methods:**

Non-targeted metabolomic profiling using ¹H-NMR spectroscopy was applied to hydrophilic extracts of the three cell lines. Multivariate statistical analyses (PCA and PLS-DA) were used to identify discriminating metabolites, and pathway analysis was performed to determine altered metabolic networks.

**Results:**

Several metabolic pathways were significantly altered in melanoma cells compared to fibroblasts, including starch and sucrose metabolism, alanine, aspartate and glutamate metabolism, and glutathione metabolism. Metabolites showing more than two-fold differences included elevated UDP-glucose, ATP, glycerophosphocholine, GTP, creatine and glutathione in the melanoma cells, and reduced glucose, glutamine and 1-methylnicotinamide in fibroblasts. Comparison of the metabolites of melanoma cell lines with differing metastatic potential revealed changes in taurine and hypotaurine, β-alanine-, glutathione-, and amino acid metabolism. Metabolites showing the largest concentration changes were UDP-glucose, glutathione, NAD^+^, alanine and β-alanine.

**Conclusion:**

Metabolomic profiling revealed distinct metabolic reprogramming between melanoma and normal fibroblasts, characterized by enhanced glycolysis and glutathione-dependent antioxidant defense. Highly metastatic melanoma cells demonstrated stronger redox adaptation and altered amino acid utilization, with elevated glutathione and glutamate and reduced NAD⁺ and pyruvate, indicating a metabolic shift toward oxidative stress resistance.

## Introduction

Melanoma, also known as malignant melanoma or cutaneous melanoma, is a type of cancer that originates in melanocytes [1]. It is estimated that there will be over 110,000 new melanoma cases in the US in 2026, accounting for around 5.3% of all cancer cases. Epidemiological data indicate that the 5-year relative survival rate for non-metastatic, localised melanoma is over 99%. However, this drops to 76% for regional metastatic cases and 35% for distant metastatic cases [2]. Despite ongoing, intense research into metastasis, anti-metastatic therapies are either lacking or show limited success [3]. Therefore, a deeper understanding of how metastatic cancer cells work is still required.

Tumor cells generate energy primarily through glycolysis, rather than oxidative phosphorylation and the tricarboxylic acid (TCA) cycle, even when there is sufficient oxygen present (Warburg effect) [4]. Although glycolysis produces smaller amount of ATP than oxidative phosphorylation (OXPHOS), it provides energy more quickly to meet the high energy demands of rapidly proliferating cells [5]. Metastasizing cancer cells dynamically adapt their metabolism to the changing environment during the metastatic cascade, which is crucial for their survival [6]. In metastatic cancer, the transcription factors HIF-1α (hypoxia-inducible factor 1 alpha) and c-Myc play an important role in the upregulation of many enzymes involved in glucose metabolism. These include serum lactate dehydrogenase (LDH), which catalyzes the conversion of pyruvate to lactate, resulting in high lactate levels in cancer cells. Elevated LDH is associated with a poor prognosis in melanoma and several other cancers [7–10]. However, further progression requires increased mitochondrial function, as well as increased glutaminolysis and fatty acid oxidation [11]. In addition to being an energy source, glutamine is a precursor for the synthesis of glutathione, which is essential for maintaining redox homeostasis. It also serves as a source of carbon and nitrogen for the biosynthesis of amino acids, nucleotides, and lipids [12,13]. The metabolism of several other amino acids is also disturbed in metastatic cells. Kim et al. observed a progressive increase in several amino acids, including glycine, pyroglutamic acid, cysteine, ornithine, tyrosine and lysine, as the metastatic potential of melanoma cell lines increased [14].

As changes in metabolite levels are a molecular consequence of changes in cellular activity, metabolic profiling is an important tool for discovering biomarkers that can be used to diagnose tumor progression [15–18]. Of the analytical techniques used to monitor metabolic changes in different types of cancer, HPLC-MS, GC-MS and NMR spectroscopy have been employed [19–21]. As each platform has its own advantages and disadvantages, they are considered complementary techniques in metabolomics research. LC–MS/MS-based analytical approaches are an integral component of contemporary clinical and translational oncology, particularly with regard to metabolite quantification and pharmacological monitoring [22,23]. LC-MS is highly sensitive, making it suitable for analysing low-concentration metabolites. In contrast, high-field and high-resolution NMR spectroscopy offers lower sensitivity but higher reproducibility. Moreover, NMR-based metabolomics has several additional advantages, including its non-destructive nature, minimal sample preparation requirements, and inherently quantitative signal response. Furthermore, NMR offers robust structural information, facilitating reliable metabolite identification. These features make NMR particularly suitable for comparative metabolic profiling, despite the higher sensitivity typically achieved by MS-based approaches [24]. For the evaluation of NMR data, different multivariate statistical methods are complementarily needed. Unsupervised principal component analysis (PCA) is commonly used as an exploratory method to detect potential outliers and visualize clustering patterns in datasets. Partial least squares (PLS) regression, particularly when combined with discriminant analysis (PLS-DA), identifies and models the metabolite patterns that most effectively distinguish between predefined groups, focusing on variation directly related to class differences. The importance of the variables can be assessed using variable importance in projection (VIP) scores, which show how much each variable contributes to the model [25].

Despite increasing knowledge of metabolic reprogramming in melanoma, the relationship between metabolic alterations and metastatic potential remains incompletely understood. While several studies have identified metabolic differences between cancerous and non-cancerous cells, fewer have systematically compared melanoma cell lines with distinct metastatic capacities under controlled conditions [14,26–30]. Moreover, the metabolic characterization of specific melanoma models such as HT168-M1 and WM983B remains limited, particularly using high-resolution ^1^H-NMR-based metabolomics.

Therefore, the aim of the present study was to characterize the metabolic profiles of melanoma cell lines with high (HT168-M1) and low (WM983B) metastatic potential, and to compare them with normal human fibroblasts (MRC-5) as a physiological reference. Fibroblasts are increasingly recognized as metabolically active and highly responsive cells that regulate tissue homeostasis through interconnected signaling and mechanobiological pathways, and their established role in modulating tumor metabolism and the microenvironment further supports their suitability as physiologically relevant controls in cancer metabolomics studies [31,32]. The WM983B and HT168-M1 cell lines are both derived from lymph node metastases, but the former has much lower metastatic potential. The HT168-M1 human melanoma cell line is the derivative of the A2058 cell line and has an extremely high liver-colonizing capacity [33–36]. Both cell lines express the BRAF V600E mutation and thus exhibit increased HIF-1α expression.

By applying ^1^H NMR-based metabolomics and multivariate statistical analysis, we sought to identify key metabolites and pathways associated with metastatic behavior, thereby contributing to a better understanding of melanoma progression and potential metabolic vulnerabilities.

## Materials and methods

### Cell cultures and cell collections

The HT168-M1 human melanoma line (RRID: CVCL_2H39) is derivatives from the A2058 cell line [35] with in vivo selection for high liver metastatic capacity. WM983B (RRID: CVCL_6809) melanoma cell lines were gifts from M. Herlyn (Wistar Institute, Philadelphia, PA). MRC-5 (RRID: CCL-171) was obtained from ATCC. The human melanoma cells were cultured in RPMI-1640 culture media (Sigma-Aldrich Ltd., Budapest, Hungary), and the MRC-5 human fibroblast cells were grown in DMEM (Dulbecco’s modified Eagle’s medium, Sigma-Aldrich Ltd., Budapest, Hungary) culture media with 10% FBS (Fetal bovine serum) and 1% Penicillin/Streptomycin. The culture medium was removed from the culture flasks (4 pieces of confluent T-75 flask per cell line), then cells were washed up quickly twice with ice-cold PBS (phosphate buffered saline). The residual PBS was removed by vacuum. Then, 3 ml of ice-cold methanol was added to the cells, and cell-scraper was used for cell collection. The methanol, containing the cells was pipetted into a 15 ml centrifuge tube. To ensure statistical reliability, biological replicates were prepared from each cell line. A total of 43 cell cultures were produced, comprising 16 HT168-M1, 16 WM983B, and 11 MRC5 cultures. All cells were cultured in the Department of Experimental Pharmacology, National Institute of Oncology, Budapest, Hungary.

### Extraction of metabolites

The quenched cells were suspended in methanol, frozen in liquid nitrogen, and then thawed using a sonicator water bath (Bransonic Ultrasonic cleaner, Branson 1510-MT, Branson Ultrasonic corp., Danbury, CT, US) at room temperature to rupture the cell membrane and release metabolites from the cytosol. The freeze-thaw cycle was repeated three times. The polar metabolites were extracted from the cultured cells using a mixture of methanol, chloroform and water in a ratio of 10:10:9, with a volume of 15 ml. The same sonicator was used as before to mix the solvents. In addition, the mixtures were vortexed for 30 seconds using a classic vortex mixer (Velp Scientifica Srl, Usmate, Italy). The phases were then separated by centrifugation at 10,000 x g for 10 minutes using Sigma 2-16P benchtop centrifuge (Sigma Laborzentrifugen GmbH, Germany). After centrifugation, the aqueous/methanol phase containing the polar metabolites was transferred to 15 ml centrifuge tubes, and most of the solvent was evaporated under a stream of nitrogen. The remaining solvent was then removed by freeze drying (Scanvac CoolSafe 55−4 LaboGene ApS Lynge, Denmark).

### NMR measurements

The extracted and dried material was dissolved in 800 μL of D_2_O (99.96% D, VWR International Ltd., Budapest, Hungary), and 20 μL of a tenfold diluted TSP solution (0.75 wt.% trimethylsilyl propionic acid sodium salt, 99.9% D, Sigma-Aldrich Ltd., Budapest, Hungary) was added as a chemical shift reference standard. To prevent bacterial growth, 0.2 mg of sodium azide (Sigma-Aldrich Ltd., Budapest, Hungary) was used. The pH of the final solutions was adjusted to 7.00 ± 0.02 using diluted DCl and NaOD solutions, with the pH being measured using an Orion Star A211 benchtop pH meter (Thermo Fisher Scientific Inc., Waltham, MA, US) equipped with a Metrohm 6.0204.100 combined pH glass electrode, which was calibrated by NBS (National Bureau of Standards) buffer solutions. The solution was then centrifuged at 10,000 x g for 10 minutes using an Eppendorf MiniSpin microcentrifuge (Eppendorf AG, Hamburg, Germany). Then, 650 μL of the supernatant was transferred to a 5 mm NMR tube. NMR spectra were recorded at 25 °C using a Varian VNMRS spectrometer (Varian Inc., Palo Alto, USA) operating at a ^1^H frequency of 599.9 MHz. In the ^1^H-NMR experiments, presaturation of the residual water signal was applied. The spectra were obtained using the following acquisition parameters: a 90° flip angle, a relaxation time of 4.7 s, a spectral window of 7184 Hz, 32 K data points and 1024 scans.

### NMR spectra processing and data transformation

NMR spectra were processed using the ACD NMR Processor Academic Edition 12.01 software (Advanced Chemistry Development Inc., Toronto, ON, Canada). The FIDs were digitized to 64k data points, and an exponential multiplication with a line broadening factor of 0.5 Hz was used. Following manual phase correction, automatic baseline correction (3rd order polynomial) was applied, and the spectra were referenced to TSP (0.00 ppm). Integration of the spectra was performed in 0.02 ppm bins from 0.8 ppm to 9.4 ppm excluding the region of the water signal (4.7–4.9 ppm).

^1^H-^1^H 2D TOCSY spectra were also recorded for the melanoma cell line extracts, using 100 ms mixing time, 80 transients and 256 increments. The spectra were digitized to 2048x2048 data points.

### Statistical analysis

The statistical analysis of the NMR data was performed using Metaboanalyst 6.0 [37].

The binned data from the ACD NMR Processor program were extracted as a csv (comma separated value) file. A matrix containing data of 11–16 biological replicates of three different cell types was then built and imported into Metaboanalyst. First, 10% of the data was filtered out using the interquantile region variance filter. Then, the data were normalized using the sum of integrals, and autoscaling was applied to obtain a Gaussian distribution of the data [38]. Multivariate data analyses, including PCA and PLS-DA were performed on the data series, pairwise. Outliers were detected using PCA score plots of the first two PCs. The PLS-DA score plots of the first two components were then created to visualize the discrimination between the cell lines. The PLS-DA score plots were also generated, incorporating the binned data from all three cell lines. Finally, the VIP score plots of the first fifteen most important features were generated. Metabolites with VIP scores greater than 1.5 were considered important for discrimination purposes. Boxplots of the metabolite concentrations were used to visualize their relative concentrations in the cell lines, and fold change values were calculated to improve the interpretability of the results. The R^2^ and Q^2^ values obtained from a ten-fold cross validation process involving five components were used to evaluate the predictive ability of the PLS-DA models.

To aid the interpretation of the statistical results, the metabolite sets obtained in the PLS-DA analysis that showed discrimination, were subjected to Metabolic Pathway Analysis (MetPA) in MetaboAnalyst 6.0, using the Kyoto Encyclopedia of Genes and Genomes (KEGG) pathway library for *Homo sapiens* [39].

## Results

### Identification of the metabolites

**Fig 1:**
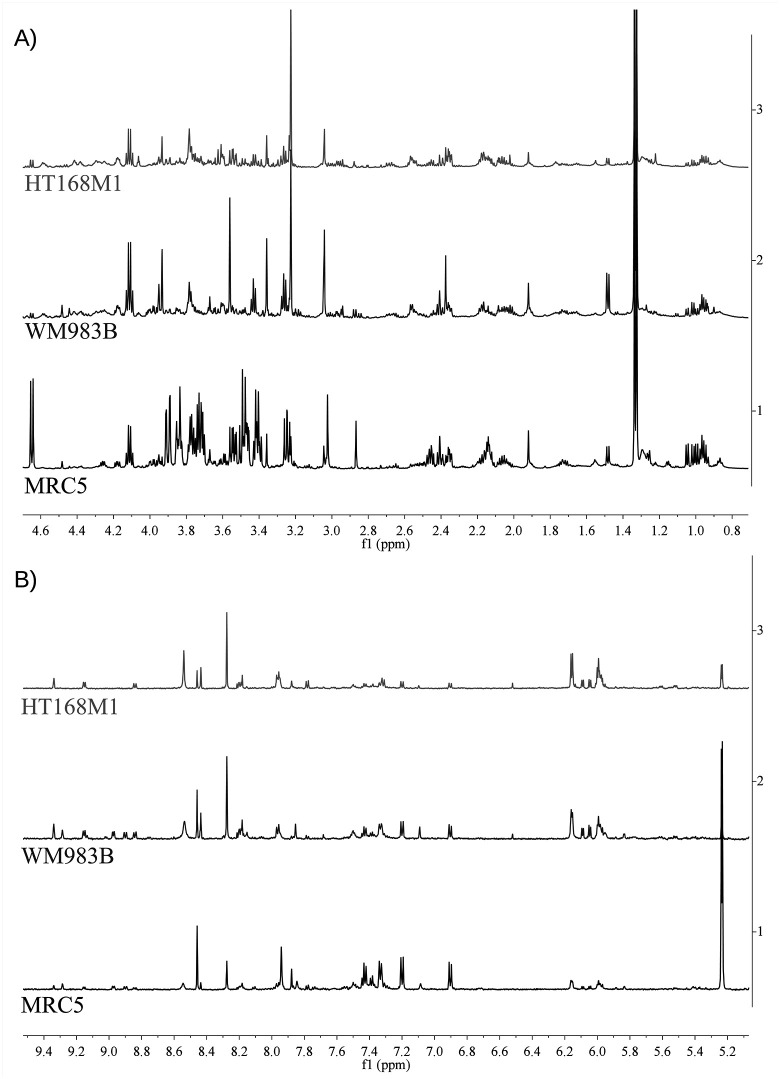
Overlayed ^1^H NMR spectra of the studied cell lines. **(A)** Aliphatic region. **(B)** Aromatic region (magnified twice compared to the aliphatic region). Y-axis: 1 – MRC5; 2 – WM983B; 3 – HT168-M1.

Fig 1 shows an example of a 1D ^1^H NMR spectrum of the extracted hydrophilic metabolites for each cell line. The spectral regions were assigned to the corresponding metabolites using the Human Metabolome Database (www.hmdb.ca) [40], as well as the recorded 2D ^1^H-^1^H TOCSY spectra of the melanoma cell extracts (S1–S3 Figs in S1 File). A total of 47 metabolites were identified; a list of these, along with their spectroscopic data, can be seen in Table 1.

**Table 1:** The ^1^H-NMR chemical shift and peak multiplicities of the metabolites identified in the NMR spectra of the studied cell lines.

Metabolite	*δ* ^1^H (ppm) / multiplicity
Acetate	1.92 s
Alanine(Ala)	1.48 d; 3.78 q
β-alanine (β-Ala)	2.55 t; 3.18 t
ATP	4.27 m; 4.41 m; 4.52 m, 4.59 t; 6.16 d; 8.28 s; 8.54 s
Arginine	1.66 m; 1.73 m; 1.92 m; 3.25 t; 3.77 t
Asparagine (Asn)	2.86 dd; 2.96 dd; 4.00 dd
Aspartate (Asp)	2.68 dd; 2.82 dd; 3.90 dd
Citrate	2.53 d; 2.65 d
Creatine (Cr)	3.04 s; 3.93 s
Creatine phosphate (CrP)	3.04 s; 3.95 s
D-glucose (Gluc)	3.23 dd; 3.40 m; 3.46 m; 3.52 dd; 3.73m; 3.82 m; 3.89 dd; 4.64 d; 5.22 d
Formate	8.46 s
Fumarate	6.52 s
Glutamate (Glu)	2.06 m; 2.13 m; 2.35 m; 3.76 dd
Glutamine (Gln)	2.14 m; 2.46 m; 3.78 dd
Glutathione (GSH)	2.17 m; 2.56 m; 2.96 m; 3.78 m; 4.57 dd
Glycerophosphocholine (GPC)	3.23 s; 3.64 m; 4.31 m
Glycine (Gly)	3.56 s
GTP	5.94 d; 8.15 s
Histidine (His)	3.14 dd; 3.23 dd; 3.99 dd; 7.09 s; 7.88 s
Hypotaurine	2.65 t; 3.36 t
Isoleucine (Ile)	0.94 t; 1.02 d; 1.27 m; 1.47 m; 1.98 m; 3.67 d
Lactate	1.33 d; 4.11 q
Leucine (Leu)	0.97 t; 1.72 m; 3.74 m
Lysine (Lys)	1.46 m; 1.71 m; 1.89 m; 3.04 t; 3.76 t
Methionine	2.13 m; 2.14 s; 2.20 m; 2.64 t; 3.86 dd
1-methylguanine	3.49 s; 7.88 s
1-methylnicotinamide	4.48 s; 8.19 t; 8.90 d; 8.97 d; 8.29 s
3-methyl-2-oxovalerate	0.90 t; 1.10 d; 1.47 m; 1.70 m; 2.94 m
Myo-inositol	3.29 t; 3.54 dd; 3.63 t; 4.06 t
NAD^+^	4.24 m; 4.38 m; 4.44 dd; 4.52 m; 6.05 d; 6.09 d; 8.18 s; 8.20 dd; 8.44 s; 8.84 d; 9.15 d; 9.34 s
Ornithine (Orn)	1.75 m; 1.84 m; 1.94 m; 3.06 t; 3.79 t
Phenylalanine (Phe)	3.13 dd; 3.29 dd; 3.99 dd; 7.33 m; 7.39 m; 7.43 m
O-Phosphocholine (PCho)	3.23 s, 3.60 m; 4.16 m
Proline	1.99 m; 2.06 m; 2.35 m; 3.33 m; 3.42 m; 4.13 dd
Pyruvate	2.37 s
Pyroglutamate	2.04 m; 2.40 m; 2.51 m; 4.17 dd
Serine	3.84 dd; 3.95 dd; 3.99 dd
Taurine (Tau)	3.27 t; 3.43 t
Threonine (Thr)	1.33 d; 3.59 d; 4.26 m
Tryptophan	3.32 dd; 3.50 dd; 4.06 t; 7.21 t; 7.30 t; 7.32 s; 7.55 d; 7.74 d
Tyrosine (Tyr)	3.04 dd; 3.19 dd; 3.93 dd; 6.90 d; 7.20 d
UDP-glucose	5.61 dd; 5.98 d,d; 7.96 d
UDP-N-acetylglucosamine (UDP-GlcNAc)	5.52 dd; 5.98 d,d; 7.96 d
UMP	8.11 d; 5.98 d
Valine (Val)	0.99 d; 1.05 d; 2.28 m; 3.62 d

s – singlet; d – doublet, t – triplet, q – quartet, m – multiplet, dd – doublet of doublet

### Multivariate data analysis

Statistical analysis was performed on a total of 43 cell line extracts (16 HT168-M1, 16 WM983B, 11 MRC5) using Metaboanalyst 6.0 [37]. After normalization by sum and scaling of the binned NMR data, PCA was first applied to detect possible outliers. Visual inspection of the PCA score plots, which were constructed using a pairwise analysis of the cell lines under study, revealed no outliers among the samples (S4 Fig in S1 File). Consequently, all 43 samples were included in the subsequent analysis.

**Fig 2:**
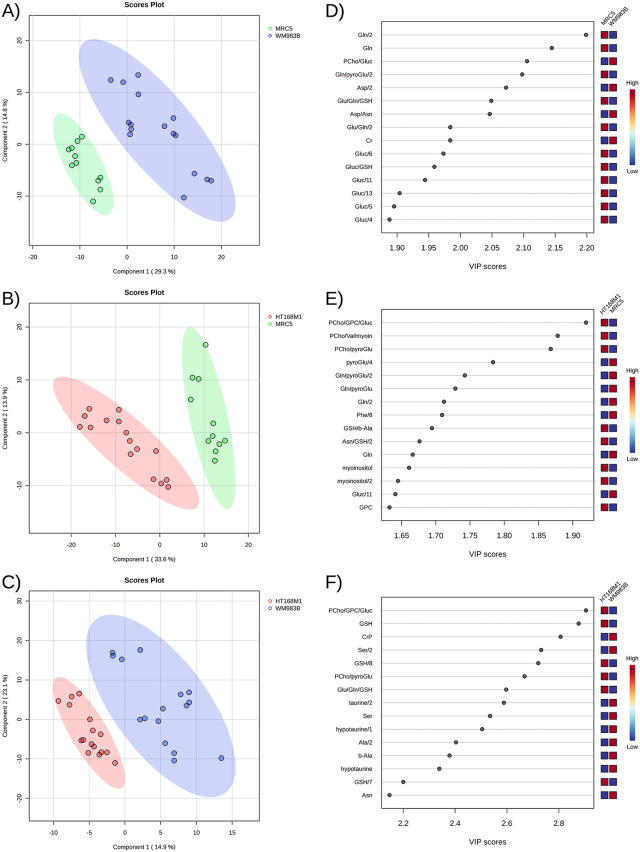
PLS-DA scores plots of the cell lines studied using pairwise analysis and VIP scores of the metabolites. **(A)** Scores plots of MRC5 and WM983B cell lines. **(B)** Scores plots of MRC5 and HT168-M1 cell lines. **(C)** Scores plots of HT168-M1 and WM983B cell lines. **(D)** VIP scores of the most important metabolites distinguishing between MRC5 and WM983B cell lines. **(E)** VIP scores of the most important metabolites distinguishing between MRC5 and HT168-M1 cell lines. **(F)** VIP scores of the most important metabolites distinguishing between HT168-M1 and WM983B cell lines.

Supervised PLS-DA analysis was used to classify the cell types in pairs. The PLS-DA scores plots clearly distinguish between the investigated cell line pairs (Fig 2A-C). PLS-DA scores plot was also created incorporating the data of all three cell lines. As can be seen in S5 Fig in S1 File, the melanoma cells are clearly distinct from one another. However, there is some overlap between HT168-M1 and MRC5, and greater overlap between WM983B and MRC5.

**Fig 3:**
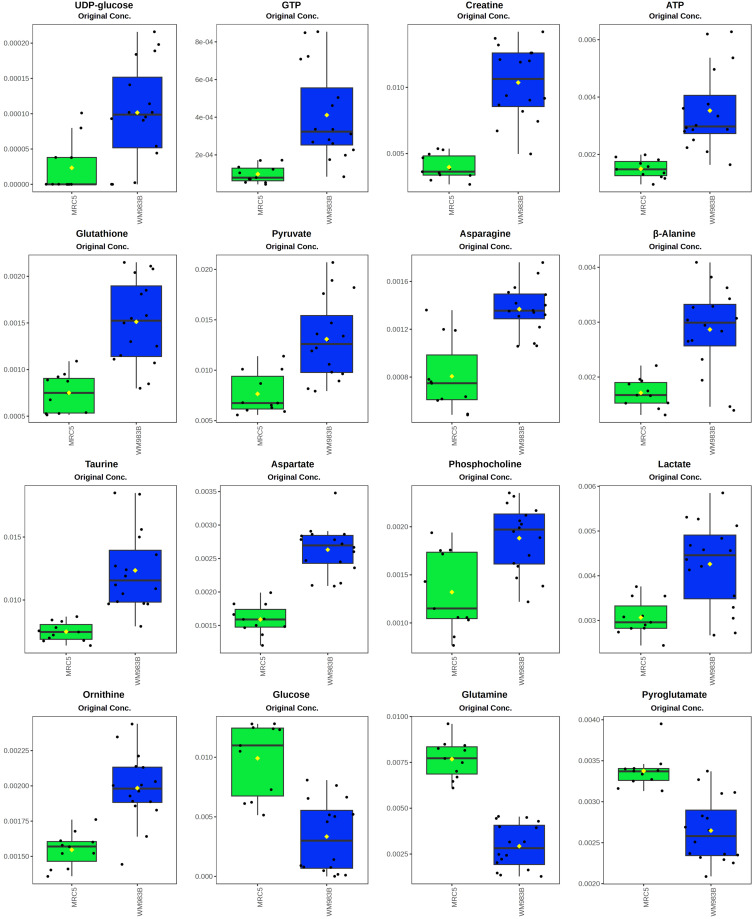
Boxplots showing concentrations of the metabolites that distinguish between the MRC5 and WM983B cell lines.

**Fig 4:**
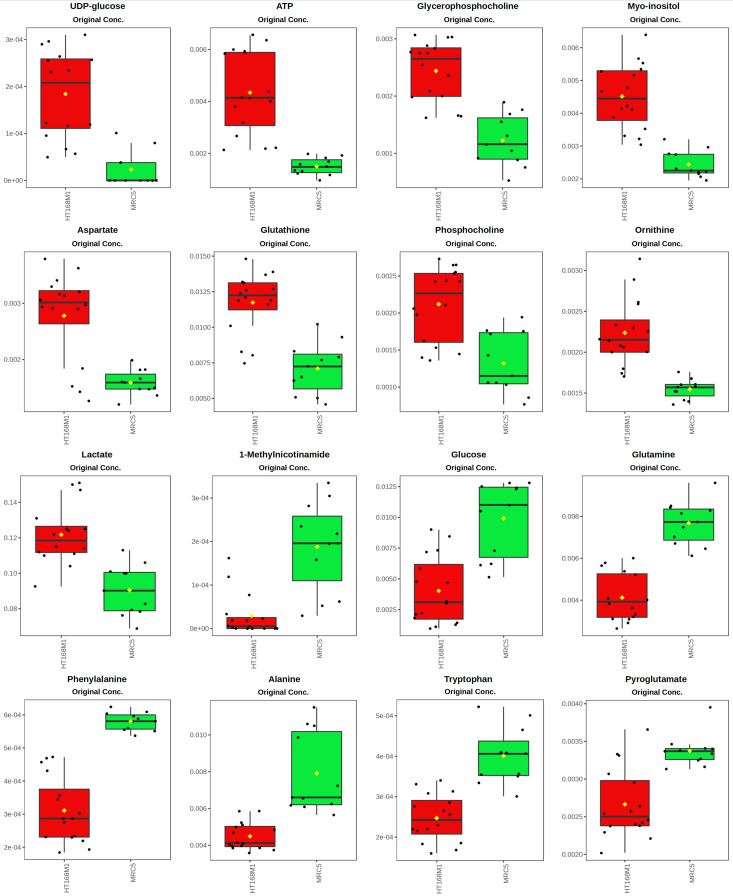
Boxplots showing concentrations of the metabolites that distinguish between the MRC5 and HP168-M1 cell lines.

**Fig 5:**
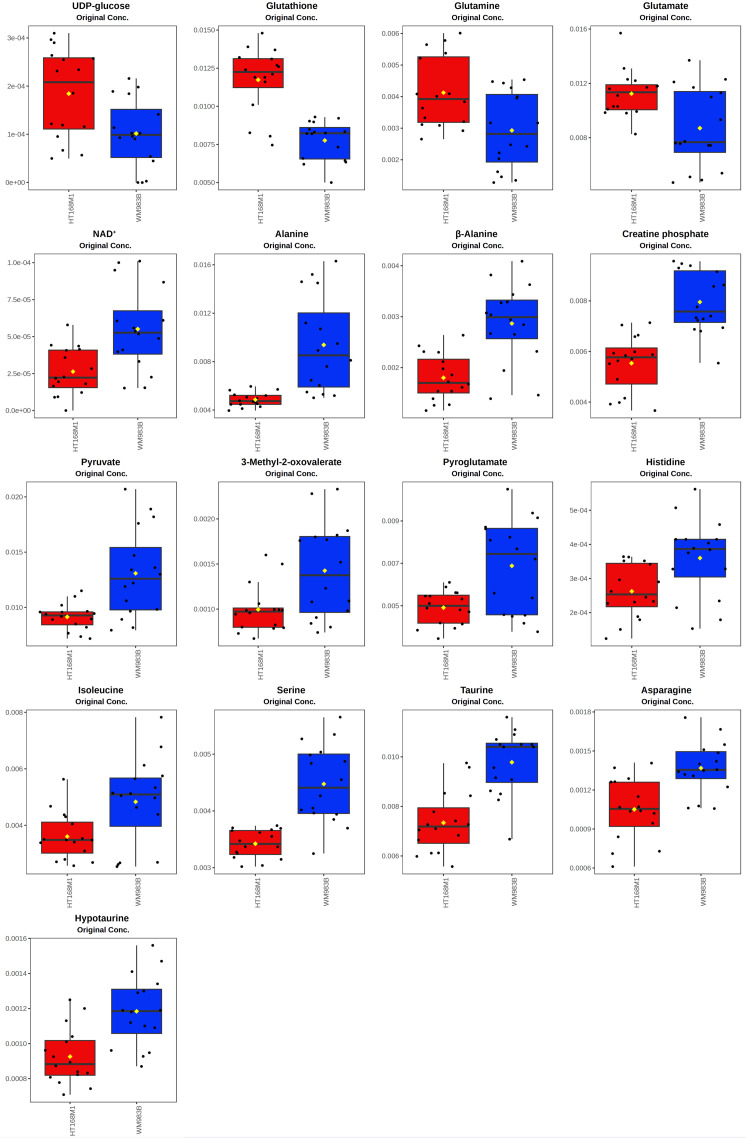
Boxplots showing concentrations of the metabolites that distinguish between the HT168-M1 and WM983B cell lines.

The VIP score plots were also generated to show the important features (Fig 2D-F), revealing the first fifteen features that distinctively characterize cell types. As most metabolites have multiple signals in the ^1^H NMR spectrum, the metabolites assigned to the 0.02 bin spectral ranges (features) often appear repeatedly. Therefore, features with VIP scores greater than 1.5 were considered significant and are listed in S1-S3 Tables in S1 file. Overlapping metabolite signals mean that a single bin may be assigned to more than one metabolite. However, the multiple appearance of metabolites and selective integration areas (S4 Table in S1 file) make it possible to identify the metabolites responsible for spectral differences. These metabolites are listed in Tables 2–4, along with their fold change in concentration. The box plots of concentrations for the most important metabolites are shown in Figs 3–5.

**Table 2:** Fold change of concentrations (WM983B/MRC5) of the key metabolites that distinguish between the MRC5 and WM983B cell lines.

Metabolite	fold change
UDP-glucose	4.51
GTP	4.34
Creatine	2.67
ATP	2.43
Glutathione	2.07
Pyruvate	>1.74*
Asparagine	1.73
β-Alanine	1.71
Taurine	1.70
Aspartate	1.69
Phosphocholine	1.45
Lactate	1.42
Ornithine	1.31
Glucose	0.34
Glutamine	0.39
Pyroglutamate	0.80

* The only singlet signal of pyruvate overlaps with the multiplet of glutamate. However, since the specific integration range of glutamate has a fold change value of 0.82, the real fold change for pyruvate must be greater than the value obtained for the overlapping area.

**Table 3:** Fold change of concentrations (HT168-M1/MRC5) of the key metabolites that distinguish between the MRC5 and HT168-M1 cell lines.

Metabolite	fold change
UDP-glucose	8.11
ATP	2.98
Glycerophosphocholine	2.09
Myo-inositol	1.90
Aspartate	1.79
Glutathione	1.69
Phosphocholine	1.65
Ornithine	1.49
Lactate	1.39
1-Methylnicotinamide	0.15
Glucose	0.42
Glutamine	0.55
Phenylalanine	0.55
Alanine	0.59
Tryptophan	0.63
Pyroglutamate	0.79

**Table 4:** Fold change of concentrations (HT168-M1/WM983B) of the key metabolites that distinguish between the HT168-M1 and WM983B cell lines.

Metabolite	fold change
UDP-glucose	1.80
Glutathione	1.52
Glutamine	1.43
Glutamate	1.32
NAD^+^	0.48
Alanine	0.53
β-Alanine	0.64
Creatine phosphate	0.70
Pyruvate*	< 0.71
3-Methyl-2-oxovalerate	0.71
Pyroglutamate	0.73
Histidine	0.75
Isoleucine	0.76
Serine	0.77
Taurine	0.78
Asparagine	0.78
Hypotaurine	0.79

* The only singlet signal of pyruvate overlaps with the multiplet of glutamate. However, since the specific integration range of glutamate has a fold change value of 1.32, the real fold change for pyruvate must be smaller than the value obtained for the overlapping area.

To assess the predictive ability of the PLS-DA models, a 10-fold cross validation with 5 components was performed. In all cases R^2^ values were above 0.98 and Q^2^ values were above 0.88, confirming the good predictive ability of the model (MRC5 and WM983B: R^2^ = 0.994, Q^2^ = 0.891; MRC5 and HT168-M1: R^2^ = 0.994, Q^2^ = 0.895; HT168-M1 and WM983B: R^2^ = 0.986, Q^2^ = 0.885)

A comparison of the healthy MRC5 cell line and the WM983B cell line revealed that 16 metabolites were important in discriminating between the two. Higher levels of glutamine, glucose (Gluc) and pyroglutamate (pyroGlu) are seen in MRC5 and all the other metabolites listed in Table 1 are elevated in the melanoma cell line, the most important with high VIP scores being phosphocholine (PCho), aspartate and creatine (Cr) (Fig 3D). Examining the changes in metabolite concentrations between the two cell lines reveals a more than twofold decrease in glucose and glutamine concentrations in the melanoma cell line and a more than twofold increase in UDP-glucose, GTP, creatine, ATP and glutathione concentrations. A similar comparison of the highly metastatic HT168-M1 cell line with MRC5 (Table 3 and Fig 2E) shows higher levels of the same three metabolites (pyroGlu, Gln and Gluc) in MRC5 as in the comparison with the less metastatic cell line. In addition, 1-methylnicotinamide and some amino acids, such as phenylalanine, tryptophan and alanine also have higher levels in MRC5. Besides PCho which has a higher relative concentration in HT168-M1, glutathione (GSH), myo-inositol and glycerophosphocholine (GPC) seem to play the most important role in the discrimination. The highest fold-change can be observed in the concentrations of UDP-glucose, ATP, GPC, 1-methylnicotinamide and glucose.

When the two melanoma cell lines with different metastatic capacities were compared, it was found that GSH, glutamate, UDP-glucose and glutamine were present in higher proportions in the highly metastatic HT168-M1 cell line. In contrast, WM983B had higher levels of creatine phosphate (CrP), serine, taurine, hypotaurine, alanine, β-alanine and asparagine (Table 4 and Fig 2F). The differences in metabolite concentrations between the two melanoma cell lines are much more moderate than those observed in the comparison with healthy cells. Only UDP-glucose, GSH, NAD^+^, alanine and β-alanine has a fold change greater than 1.5.

### Metabolic pathway analysis

**Fig 6:**
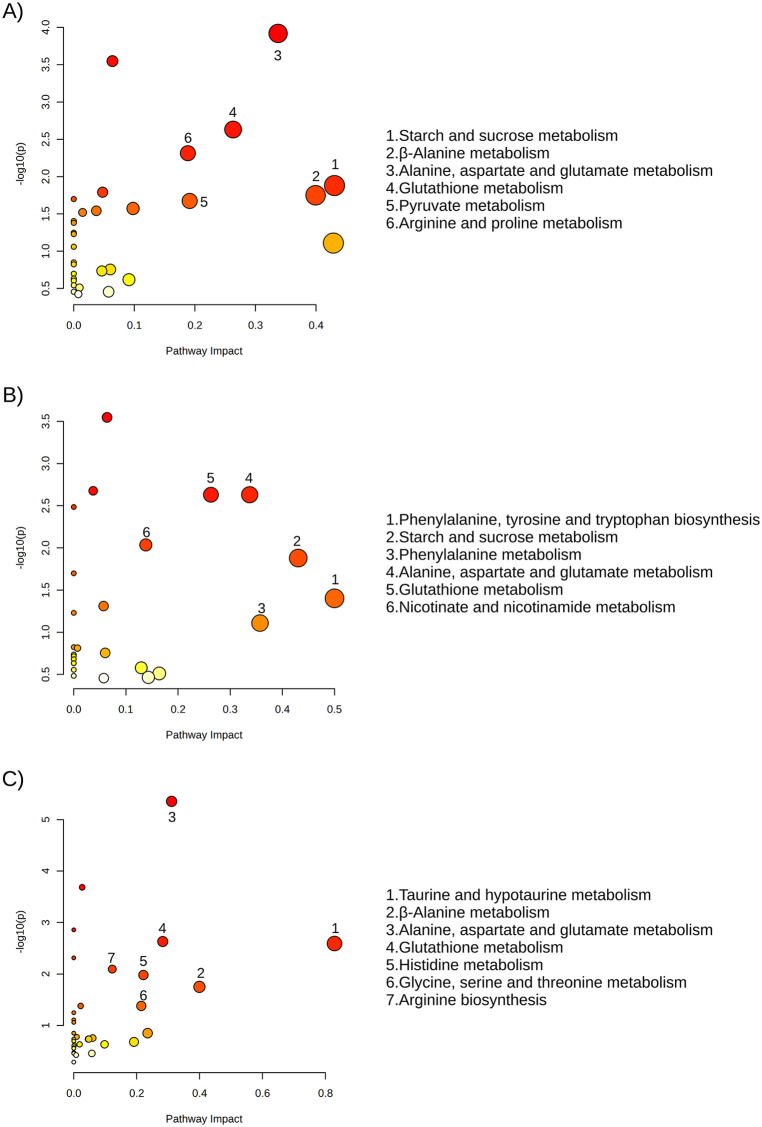
Metabolic pathway analysis. The most affected pathways with high impact (>0.1) and a *p*-value <0.05 are listed on the right. **(A)** MRC5 and WM983B cell lines. **(B)** MRC5 and HT168-M1 cell lines. **(C)** HT168-M1 and WM983B cell lines.

To facilitate interpretation of the results, the discriminating metabolite sets (Tables 2–4) were introduced into MetaboAnalyst’s metabolic pathway analysis module. The results of the analysis are summarized in the S5-S7 Tables in S1 File. The topology maps of the altered biochemical pathways are shown in Fig 6, where pathways with a high impact (>0.1) and low *p*-values (<0.05) are listed.

When the healthy MRC5 cell line is compared with melanoma cell lines, the most significant altered pathways are starch and sucrose metabolism, alanine, aspartate and glutamate metabolism, and glutathione metabolism. Additionally, the metabolism of β-alanine, pyruvate, arginine and proline are disrupted in WM983B. Meanwhile, the metabolism of aromatic amino acids, nicotinate and nicotinamide are altered in HT168-M1.

The 17 metabolites that were found to differ between the highly metastatic HT168-M1 and the less metastatic WM983B cell lines suggest alterations in the metabolism of taurine and hypotaurine, glutathione and several amino acids.

## Discussion

### Comparison of the healthy and melanoma cell lines

Examining the NMR spectra of the investigated cell lines (Fig 1), the most notable difference between the MRC5 healthy cell line and the two melanoma cell lines is the presence of multiple high-intensity glucose signals in the MRC5 spectrum in the 3.2–5.3 ppm region. Consistently, PLS-DA analysis identified glucose as a key discriminating metabolite (Table 2 and 3), reflecting the well-known increased glucose consumption of cancer cells [41]. Melanoma cells also exhibitied elevated ATP and lactate levels, indicating enhanced glycolytic activity, and confirming the aerobic glycolytic phenotype of our cell lines [5]. Lactate production acidifies the tumor microenvironment and promotes extracellular matrix degradation, angiogenesis, and immune evasion, all of which are key processes that facilitate invasion and metastatic spread. Furthermore, lactate acts as a signalling molecule that reprograms gene expression and activates pathways (e.g., GPR81, HIF-1α–associated signalling) that drive epithelial–mesenchymal transition, immune suppression, and metastatic colonization [42].

Glutamine and pyroglutamate were more abundant in MRC5 cells. Besides glucose, glutamine is the most important nutrient for cancer cells. It also serves as a source of carbon and nitrogen for the biosynthesis of amino acids, nucleotides, and lipids which are necessary for cell growth and rapid proliferation. Additionally, glutamine plays a key role in maintaining redox homeostasis by promoting the biosynthesis of GSH and NADPH [12,13]. Pyroglutamic acid (also known as 5-oxoproline or pidolic acid) is an intermediate in glutathione metabolism and has been proposed as a potential biomarker for pancreatic and breast cancers [43,44]. As expected, the levels of pyroglutamate in our comparisons are inversely related to those of GSH, an important discriminating metabolite that is upregulated in both WM983B and HT168-M1.

PCho, a key component in membrane synthesis, was also found to be upregulated in our two melanoma cell lines, with a concentration change of approximately 1.5-fold (Table 2 and 3). Elevated levels of PCho were found in most cancer types such as skin-, breast-, ovarian- and prostate cancers [21,45–47], however opposite changes are also reported for a type of lung cancer (NSCLC) [48]. A higher concentration of this metabolite results from increased choline transport into the cells and the overexpression of choline kinase. This contributes to tumor progression and metastasis [49].

There are two amino acids among the metabolites that have elevated levels in both WM983B and HT168-M1, aspartate and ornithine. Aspartate plays a crucial role in the metabolism of cancer cells because it is involved in several vital biochemical processes, such as nucleotide- and protein synthesis, and redox homeostasis, all of which are essential for cells that are proliferating rapidly [50]. Because tumor cells often have difficulty taking up aspartate from their environment, they rely on the mitochondrial catabolism of glutamine to obtain aspartate. Ornithine, a non-proteogenic α-amino acid is mainly used in cancer cells as a substrate for ornithine decarboxylase, a key enzyme in polyamine synthesis [51]. These polyamines such as spermidine and spermine are essential for cancer cell growth and division. Furthermore, elevated ornithine may reflect broader reprogramming of arginin metabolism and urea-cycle associated pathways, which are increasingly recognised as contributors to tumor growth and microenvironmental adaptation [52].

Finally, UDP-glucose (UDPG) is also another metabolite that has been found to be different between healthy and melanoma cell lines, with higher levels in the latter. UDPG plays a role in regulating cell migration, proliferation, and extracellular matrix modulation. UDPG is converted to UDP-glucuronic acid, a precursor for proteoglycans and glycosaminoglycans, by UDPG dehydrogenase, an enzyme that is overexpressed in several cancers [53].

In the pairwise comparisons between healthy and melanoma cell lines, a few metabolites were found to be able to discriminate between healthy and only one melanoma cell lines. In the pathway analysis comparing MRC5 and WM983B, β-alanine metabolism has the second highest impact (Fig 6A). β-Alanine is a non-essential amino acid and the precursor of carnitine, which plays an important role in energy production and fatty acid metabolism [54]. Kou et al. reported a significantly higher intracellular concentration of β-alanine in aggressive breast cancer cells than in non-cancerous cells [26].

Pyruvate metabolism and arginine and proline metabolism are also altered in the less metastatic cell line. The former includes two metabolites identified in our analysis: pyruvate and lactate. The higher levels of these metabolites in the melanoma cell line suggest enhanced glycolytic flux and/or reduced mitochondrial oxidative utilization of pyruvate, reflecting reprogrammed energy metabolism [55]. Pyruvate is also part of the arginine and proline metabolism pathway, along with ornithine and creatine. This latter metabolite plays an important role in the energy buffering system by forming phosphocreatine, which can rapidly generate ATP when needed, by the enzyme phosphocreatine-creatine kinase (CK). Given that rapid cell division requires immediate energy, it is not surprising that abnormal CK levels are a feature of several cancers [56].

Comparing the metabolites of the healthy cell line with those of the highly metastatic HT168-M1, unique disturbances are observed in the biosynthesis of the aromatic amino acids (phenylalanine, tyrosine and tryptophan), as well as in the metabolism of phenylalanine, nicotinate, and nicotinamide (Fig 6B). Phenylalanine and tryptophan levels were lower in the highly metastatic cell line. These two essential amino acids are crucial for the protein synthesis, with phenylalanine acting as a precursor to tyrosine, which in turn is a precursor to melanin, a pigment produced in melanocytes [57]. The metabolism of phenylalanine is often altered in cancer cells, particularly affecting the mTORC1 signalling pathway. This pathway is a critical regulator of cell growth, proliferation and lipid metabolism [58]. Tryptophan is an essential amino acid that plays a key role in cancer metabolism, primarily via the kynurenine pathway. In tumors of the digestive system, lower levels of tryptophan have been found to correlate with increased tumour cell migration, immune suppression and increased metastatic potential [59,60]. Altered amino acid metabolism in cancer cells can have a profound effect on the tumor microenvironment, suppressing or reprogramming immune cell function, particularly that of memory T-cells [61]. 1-Methylnicotinamide (MNA) is a metabolite of the nicotinate and nicotinamide metabolic pathway synthesized from nicotinamide via N-methylation by the nicotinamide-N-methyltransferase enzyme, which has been found to be overexpressed in several human cancers [32,62]. MNA is an immune-regulatory metabolite in human ovarian cancer that induces T-cells to secrete tumour necrosis factor alpha [63].

There are three other metabolites in Table 3 to be mentioned. Glycerophosphocholine is a choline metabolite, and its level is higher in HT168-M1, similarly to that of PCho. The glucose-derived metabolite, myo-inositol, is a precursor of phosphoinositides, which are recognized as signalling molecules in cells. The PI3K and PTEN enzymes, which regulate the level of phosphatidylinositol-3,4,5-triphosphate, are frequently mutated in cancer [64]. Finally, the non-essential amino acid alanine, can be produced directly from pyruvate by the enzyme alanine transaminase. It is used in protein synthesis and is also a glycolytic by-product that transports excess carbon from glycolysis [65]. Intensive use of this amino acid results in low levels in HT168-M1 cells.

### Comparison of the melanoma cell lines with high and low metastatic capacity

When evaluating the results obtained from comparing the metabolites from the two melanoma cell lines, two pathways were found to be important in all the pairwise comparisons: glutathione metabolism, and alanine, aspartate and glutamate metabolism (Fig 6). The former includes higher levels of GSH and glutamate, and lower levels of pyroglutamate in the highly metastatic cell line (Table 4). This highlights the importance of maintaining high antioxidant levels in the highly metastatic cell line, in order to cope with the reactive oxygen species produced by intense metabolic activity. These metabolic adaptations may be linked to broader pathway-regulated transcriptional programs, since AP-1-associated signaling enhance cellular antioxidant defense by upregulating genes involved in glutathione synthesis, and also in amino acid transport and metabolism [66]. An earlier study by Carretero et al. demonstrated that B16 melanoma cells with a higher GSH content exhibited greater metastatic activity in the liver than cells with a low GSH content [67]. Liver-metastatic breast cancer cells also exhibit upregulated GSH metabolism [68]. GSH has importance in key signal transduction reactions as a controller of cell differentiation, proliferation, apoptosis, ferroptosis and immune function [69]. As glutamate is a precursor of GSH, an elevated level of glutamate also supports GSH synthesis and, consequently, the oxidative stress resistance of cancer cells. In metastatic renal cell carcinoma and hepatocellular carcinoma, elevated glutamate levels have been associated with increased glutaminase activity [27,70].

The metabolites of the alanine, aspartate and glutamate metabolism pathway that distinguish the two melanoma cell lines are: alanine, pyruvate, asparagine, glutamate and glutamine. Due to the high energy demand and intensive protein synthesis, the levels of both alanine and pyruvate are lower in the highly metastatic cell line than in the low metastatic cell line. Asparagine is essential for cancer growth and development, and is a necessary component of the proteins involved in the epithelial-mesenchymal transition which promotes metastatic processes [71]. The lower level of asparagine in the HT168-M1 cell line indicates more intense metabolism of this amino acid than in the less metastatic cell line. Glutamine was found to discriminate between healthy and melanoma cell lines, as well as between melanoma cell lines with high and low metastatic capacity. The higher level of glutamine in HT168-M1 suggests that this cell line relies less on glutamine for energy production.

The pathway analysis comparing the two melanoma cell lines revealed that taurine and hypotaurine metabolism had the greatest impact, despite these metabolites having a lower fold change in concentration. WM983B has higher levels of taurine and hypotaurine relative to MRC5. Hypotaurine is an intermediate in the biosynthesis of taurine and produced by the enzyme cysteamine dioxygenase, which plays an important role in protecting against oxidative stress. Earlier studies have shown that this metabolic pathway is disrupted in several types of cancer [72–74]. Gao et al. found a strong positive correlation between glioma grade and hypotaurine levels, and demonstrated that hypotaurine activates hypoxia signaling [75]. Taurine, the oxidation product of hypotaurine, can act as a direct antioxidant by reducing the reactive oxygen species and increasing the activity of certain antioxidant enzymes [76,77].

In all the pairwise analyses, UDP-glucose is a discriminating metabolite, with levels increasing from the healthy cell line to the highly metastatic HT168-M1 cell line. In addition to the metabolites discussed so far, the level of some amino acids such as serine, isoleucine and histidine differ in the two melanoma cell lines, having higher levels in the less metastatic WM983B.

Creatine phosphate is an important metabolite that distinguishes the two melanoma cell lines from each other, as it stores energy that can be released quickly. The highly metastatic cell line has lower levels of creatine phosphate due to its intensive energy use. Levels of NAD^+^ are also low in HT168-M1. It is an essential metabolite in oxidation-reduction reactions during energy production [78]. Alterations in NAD^+^ levels may reflect shifts in cellular redox balance and mitochondrial metabolism; however, the concurrent lower pyruvate and higher GSH levels suggest redox imbalance. 3-Methyl-2-oxovalerate is the product of incomplete breakdown of branched-chain amino acids, and its level is elevated in the low metastatic cell line.

The metabolic biomarkers identified in this study may also have translational relevance, as emerging nanoparticle-based precision oncology approaches increasingly aim to exploit tumor-selective metabolic vulnerabilities [79].

Previous studies of melanoma cells derived from the primary (WM-115) and metastatic (WM-226-4) sites of the same patient identified glycerophospholipid metabolism as the most affected pathway, followed by the carnitine shuttle, which has a biological implication in the survival of metastatic cells. However, it was also found that tryptophan and branched-chain amino acid metabolism were altered [80]. Kosmopoulou et al. investigated the same two cell lines, highlighting the critical roles of purine, pyrimidine and amino acid metabolism in human melanoma metastasis [30]. Another study of melanoma cells with different metastatic potential revealed an accumulation of organic acids, particularly lactic acid, in melanoma cell lines compared to melanocytes. This accumulation increased progressively with increasing metastatic potential. Additionally, elevated levels of glucose-6-phosphate and several amino acids, including glycine, pyroglutamic acid, cysteine, ornithine, tyrosine and lysine, were found in the highly metastatic cell lines. However, the most significant affected pathway was alanine, aspartate and glutamate metabolism [14].

The metabolic pathway alterations identified in our study are in agreement with earlier findings. The apparent differences can be attributed to the different cell lines tested or the different analytical techniques employed.

## Conclusion

Our metabolomic analysis revealed distinct metabolic reprogramming between healthy fibroblasts and melanoma cell lines, as well as between melanoma cells of low and high metastatic potential. The findings highlight enhanced glycolysis and glutathione-dependent antioxidant defense as key features of melanoma metabolism, with the highly metastatic cells displaying stronger redox adaptation and altered amino acid utilization. Elevated glutathione and glutamate, together with reduced NAD⁺ and pyruvate levels, indicate a metabolic shift favoring oxidative stress resistance and survival under high energy demand. However, as this study was limited by the use of only two melanoma cell lines and in vitro conditions, future research integrating larger cell cohorts, multi-omics approaches, and in vivo validation will be essential to confirm and extend these findings.

## Supporting information

S1 File Supporting Figures and Tables. Assignation of metabolites on the ^1^H-NMR spectrum of a HT168M1 cell extract (S1 Fig.); The ^1^H-^1^H TOCSY NMR (600 MHz) spectrum of the melanoma cell line extracts (S2-S3 Figs); PCA scores plots of the cell lines studied using pairwise analysis (S4 Fig.); PLS-DA scores plots of the three cell lines studied (S5 Fig.); VIP scores by component 1 in the PLS-DA analysis of the investigated cell lines (S1-S3 Tables); Selective integration regions of the most important metabolites (S4 Table); Pathway analysis results (S5-S7 Tables).
(PDF)

S2 File Binned NMR data of the studied cell extracts. (XLSX)
