# Supplementary material for: Metabolic profiling of human melanoma cell lines with high and low metastatic capacity by 1H-NMR spectroscopy
Source: PLoS One. 2026 Jul 1;21(7):e0352639. doi: 10.1371/journal.pone.0352639 (PMC13322499; doi:10.1371/journal.pone.0352639)
Supplement: S1 File — Assignation of metabolites on the 1H-NMR spectrum of a HT168M1 cell extract (S1 Fig.); The 1H-1H TOCSY NMR (600 MHz) spectrum of the melanoma cell line extracts (S2-S3 Figs); PCA scores plots of the cell lines studied using pairwise analysis (S4 Fig.); PLS-DA scores plots of the three cell lines studied (S5 Fig.); VIP scores by component 1 in the PLS-DA analysis of the investigated cell lines (S1-S3 Tables); Selective integration regions of the most important metabolites (S4 Table); Pathway analysis results (S5-S7 Tables). (PDF) [file pone.0352639.s001.pdf]

Metabolic profiling of human melanoma cells with high and low metastatic capacity by ^{1}H-NMR spectroscopy

Nima Rezvani Kakhki[1,2], Zita Hegedűs[3], József Tóvári[4], Arash Mirzahosseini[1,2], Béla Noszál[1,2], Márta Kraszni[1,2*]

[1] Department of Pharmaceutical Chemistry, Semmelweis University, Budapest, Hungary
[2] Center for Pharmacology and Drug Research & Development, Semmelweis University, Budapest, Hungary
[3] National Korányi Institute of Pulmonology, Budapest, Hungary
[4] Department of Experimental Pharmacology, National Institute of Oncology, Budapest, Hungary

*Corresponding author

E-mail: kraszni.marta@semmelweis.hu

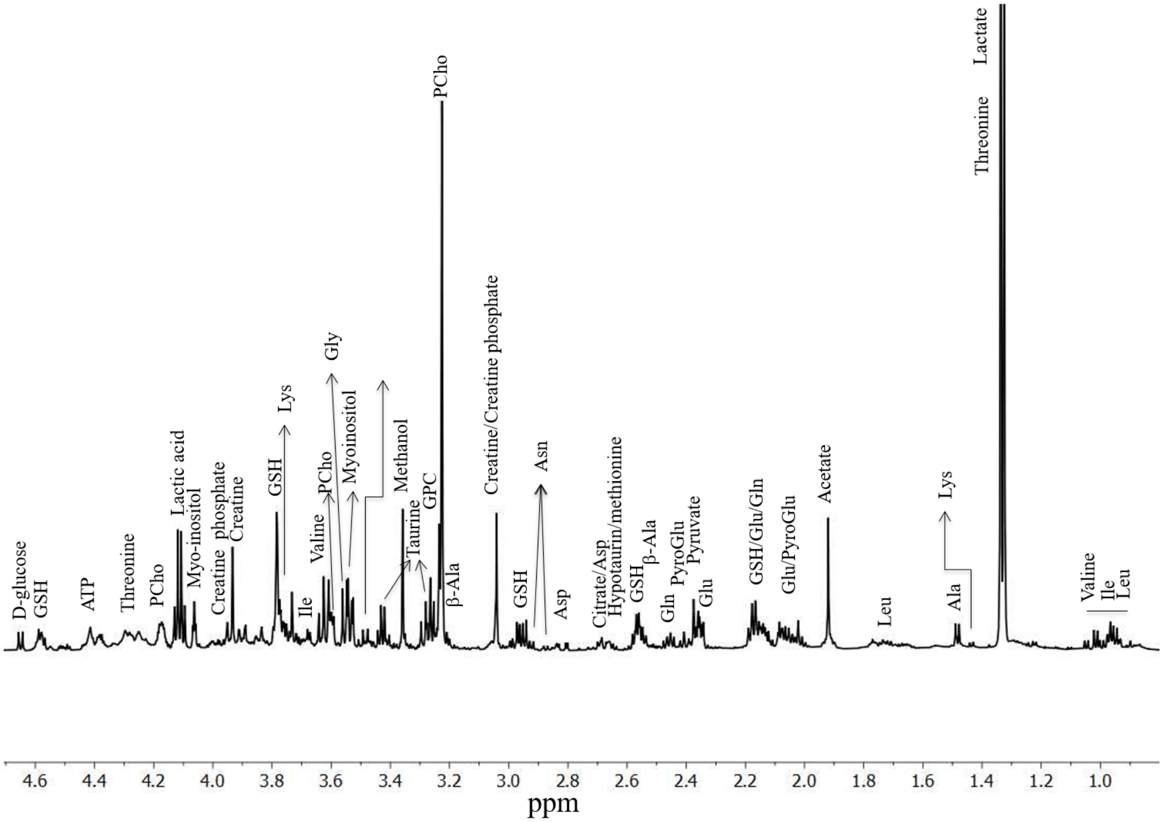

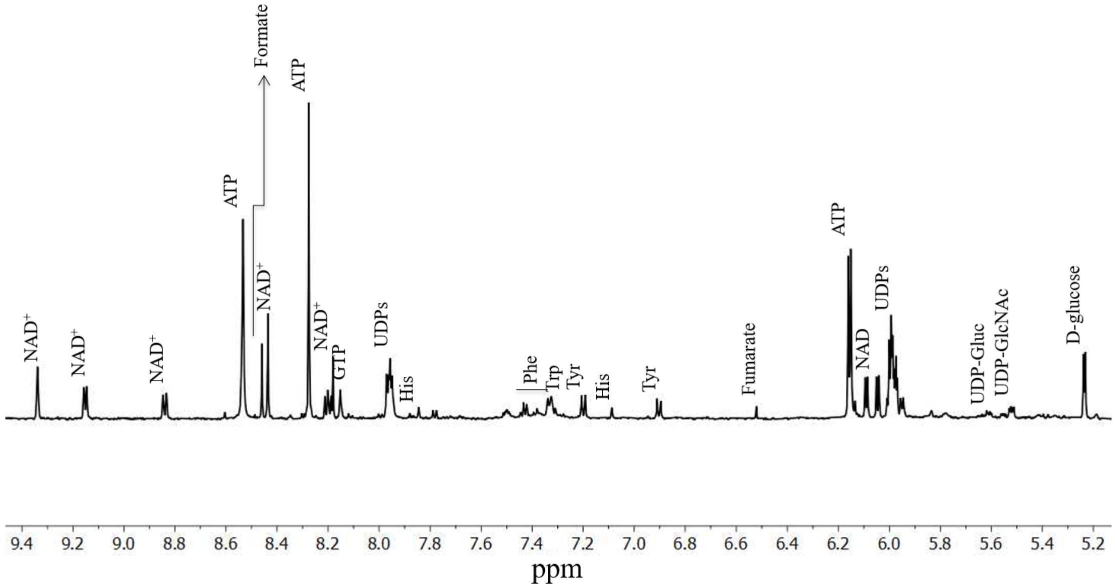

S1 Fig. Assignation of metabolites on the ^{1}H-NMR spectrum of a HT168-M1 cell extract.

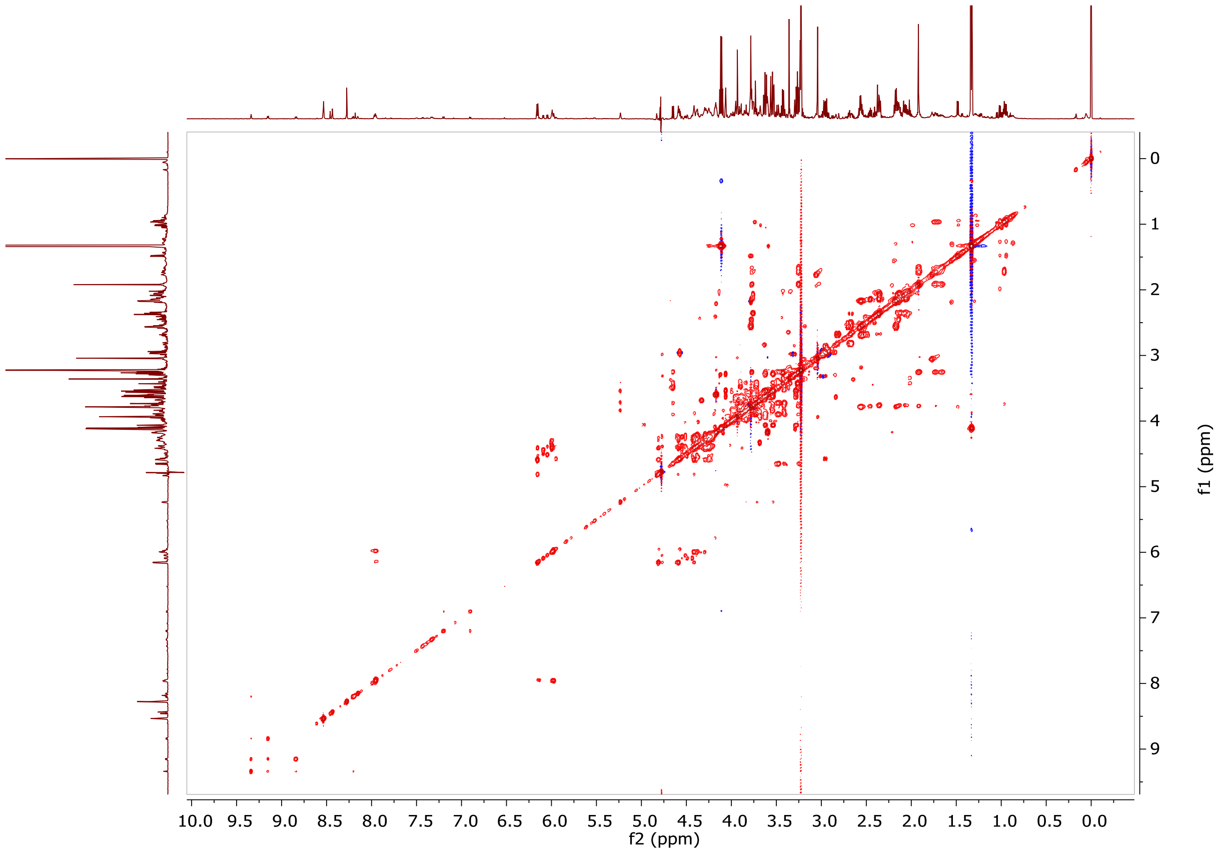

S2 Fig. The ^{1}H-^{1}H TOCSY NMR (600 MHz) spectrum of the HT168-M1 cell line extract.

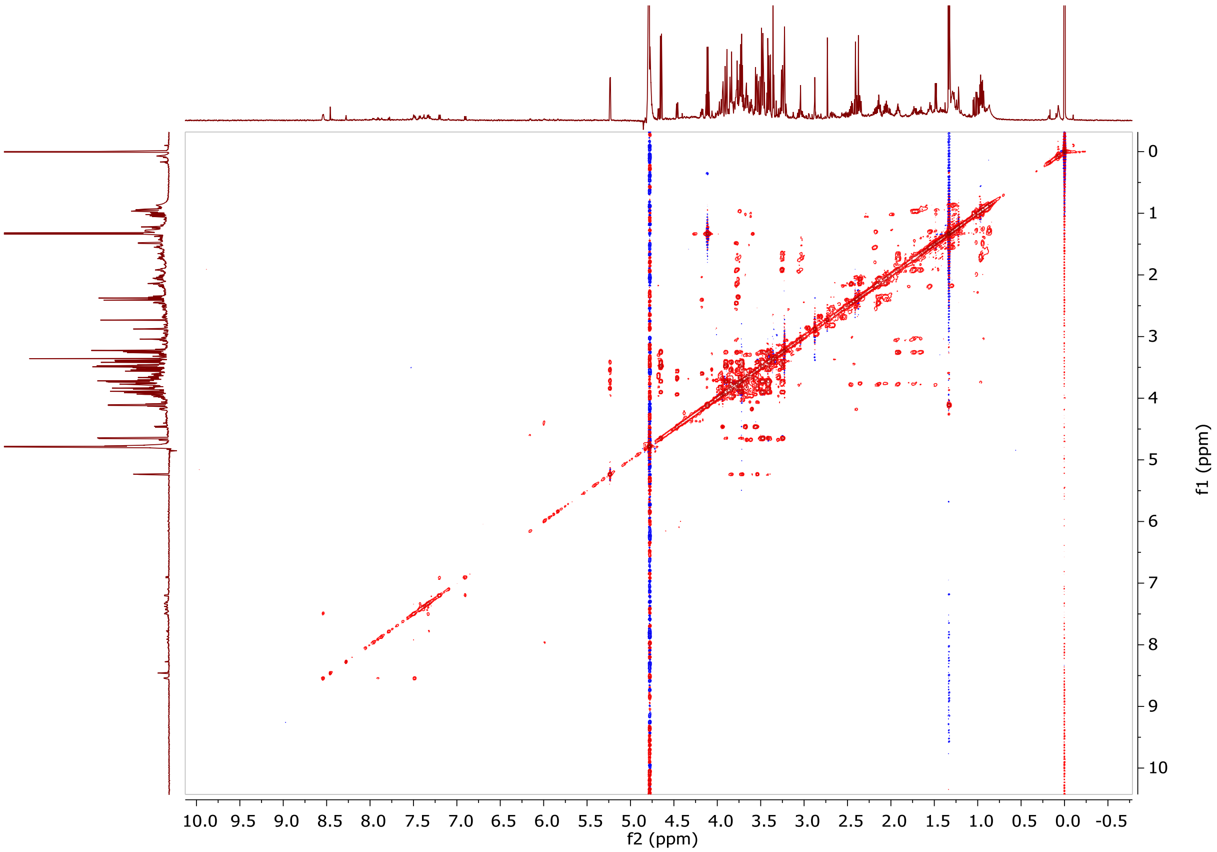

S3 Fig. The ¹H-¹H TOCSY NMR (600 MHz) spectrum of the WM983B cell line extract.

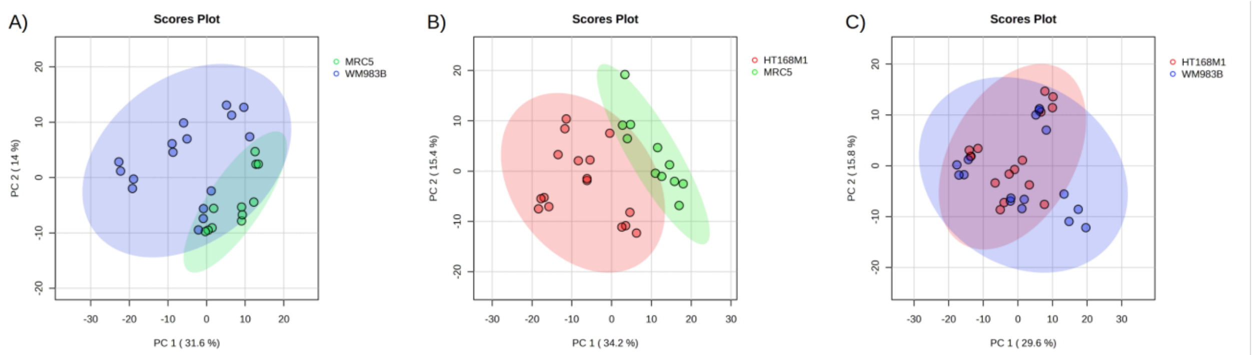

S4 Fig. PCA scores plots of the cell lines studied using pairwise analysis. (A) MRC5 and WM983B cell lines. **(B)** MRC5 and HT168-M1 cell lines. **(C)** HT168-M1 and WM983B cell lines.

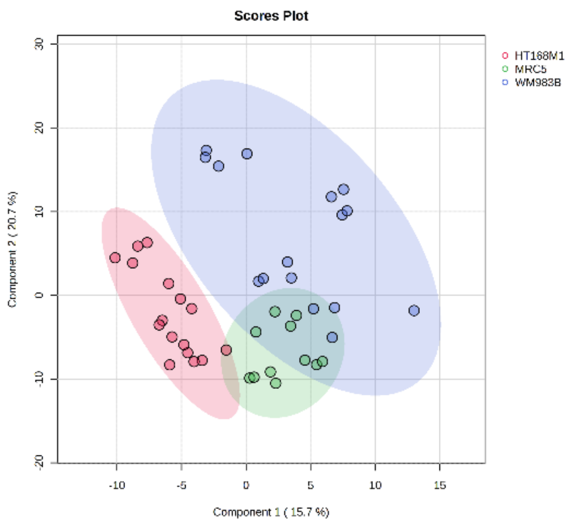

S5 Fig. PLS-DA scores plots of the three cell lines studied.

S1 Table. VIP scores by component 1 in the PLS-DA analysis of MRC5 and WM983B cell lines. The letter 'H' indicates a higher concentration.

	MRC5	WM983B	VIP score
Gln/2	H		2.1987
Gln	H		2.1446
PCho/Gluc		H	2.1055
Gln/pyroGlu/2	H		2.0978
Asp/2		H	2.0723
Glu/Gln/GSH	H		2.0489
Asp/Asn		H	2.0464
Glu/Gln/2	H		1.9840
Cr		H	1.9839
Gluc/6	H		1.9732
Gluc/GSH	H		1.9589
Gluc/11	H		1.9440
Gluc/13	H		1.9038
Gluc/5	H		1.8950
Gluc/4	H		1.8878
Gluc/14	H		1.8753
Gluc/10	H		1.8729
Asn		H	1.8651
Orn		H	1.8418
PCho/pyroGlu/2		H	1.8298
Gluc/12	H		1.8121
Gluc/8	H		1.8043
Gluc/15	H		1.8017
pyroGlu/3	H		1.7991
Gluc/2	H		1.7977
Gluc/Leu/Lys	H		1.7577
Asn/GSH		H	1.7480
Asp		H	1.7396
Cr/CrP		H	1.7368
GSH/β-Ala		H	1.7307
GSH6		H	1.7237
β-Ala		H	1.6873
Asn/GSH/2		H	1.6452
GSH/4		H	1.6291
taurine		H	1.6285
Gluc	H		1.6263
ATP		H	1.5991
Pyruvate/Glu		H	1.5989
Gluc/9	H		1.5755
Pcho/2		H	1.5678
hypotaurine		H	1.5648
GSH/9		H	1.5627
UDPs		H	1.5360
GTP		H	1.5142
lactate/2		H	1.5138

S2 Table. VIP scores by component 1 in the PLS-DA analysis of MRC5 and HT168M1 cell lines. The letter 'H' indicates a higher concentration.

Metabolites	MRC5	HT165M1	VIP scores
PCho/GPC/Gluc		H	1.9199
PCho/Val/myoinositol		H	1.8786
PCho/pyroGlu		H	1.8684
pyroGlu/4	H		1.7842
Gln/pyroGlu/2	H		1.7429
Gln/pyroGlu	H		1.7291
Gln/2	H		1.7125
Phe/8	H		1.7098
GSH/β-Ala		H	1.6949
Asn/GSH/2		H	1.6768
Gln	H		1.6668
myoinositol		H	1.6615
myoinositol/2		H	1.6454
Gluc/11	H		1.6415
GPC		H	1.6329
GSH		H	1.6291
myoinositol/3		H	1.6139
Phe/5	H		1.6108
Gluc/6	H		1.5979
Ornithine		H	1.5835
GSH/7		H	1.5767
Trp	H		1.5621
ATP		H	1.5602
Asp/Asn		H	1.5580
Asn/GSH		H	1.5500
Phe/2	H		1.5406
UDPs/4		H	1.5345
GSH/4		H	1.5324
GSH/5		H	1.5241
Gluc/10	H		1.5224
1-methylnicotinamide/5	H		1.5155
lactate/2		H	1.5104
Alanine	H		1.5103
UDPs		H	1.5090
UDP-glucose		H	1.5087
GSH/10		H	1.5051
1-methylnicotinamide/2	H		1.5005

S3 Table. VIP scores by component 1 in the PLS-DA analysis of HT168M1 and WM983B cell lines. The letter 'H' indicates a higher concentration.

Metabolites	HT168M1	WM983B	VIP score
PCho/GPC/Gluc	H		2.9049
GSH	H		2.8766
CrP		H	2.8070
Ser/2		H	2.7321
GSH/8	H		2.7207
PCho/pyroGlu	H		2.6680
Glu/Gln/GSH	H		2.5971
taurine/2		H	2.5881
Ser		H	2.5346
hypotaurine/1		H	2.5045
Ala/2		H	2.4032
β-Ala		H	2.3784
hypotaurine		H	2.3391
GSH/7	H		2.2001
Asn		H	2.1469
Pyruvate/Glu		H	2.1429
Gluc/GSH	H		2.1269
Ala		H	2.1091
Gln/pyroGlu		H	2.0189
NAD+/3		H	1.9948
pyroGlu/4 (succ/pyroGlu)		H	1.9678
Glu/3	H		1.9287
Glu/Gln/2	H		1.9151
Pro		H	1.8337
b-Ala/2		H	1.8296
GSH/5	H		1.8230
UDP-glucose/2	H		1.8072
GSH/b-Ala	H		1.7926
Gln/2	H		1.7845
3-methyl-2-oxovalerate/2		H	1.7750
Asn/GSH/2	H		1.7744
3-methyl-2-oxovalerate/Ile		H	1.7588
PCho/pyroGlu/2	H		1.7367
UDPs/4	H		1.7134
Glu/1	H		1.7095
Ile		H	1.7093
UDP-glucose	H		1.6904
Gly		H	1.6898
taurine		H	1.6831
Leu/4	H		1.6740
GSH/3	H		1.6450
Pcho (PCho/Thr)	H		1.6441
His/2		H	1.6232
3-methyl-2-oxovalerate/3		H	1.5879
Glu/Gln	H		1.5855
3-methyl-2-oxovalerate		H	1.5634
pyroGlu		H	1.5402

S4 Table. Selective intergration regions of the most important metabolites.

Metabolite	selective integration range (ppm)
Alanine	1.46-1.48; 1.48-1.50
Asparagine	2.84-2.86
Aspartate	2.66-2.68; 2.68-2.70
ATP	4.40-4.42
β-Alanine	3.18-3.20
Creatine	3.92-3.94
Creatine-phosphate	3.94-9.96
Glucose	5.22-5.24
Glutamate	2.34-2.36
Glutamine	2.46-2.48
Glutathione	2.16-2.18; 2.56-2.58
Glycerophosphocholine	4.30-4.32
GTP	8.14-8.16
Hypotaurine	2.64-2.66
Isoleucine	0.92-0.94
Lactate	1.32-1.34
1-methylnicotinamide	8.88-8.90
3-methyl-2-oxovalerate	1.08-1.10
Myo-inositol	3.28-3.30
NAD^+	8.22-8.24
Ornithine	3.06-3.08
Phenylalanine	7.42-7.44
Phosphocholine	4.14-4.16
Pyroglutamate	2.38-2.40, 2.40-2.42, 2.50-2.52
Pyruvate (Glu)	2.36-2.38
Serine	3.96-3.98
Taurine	3.26-3.28
Tryptophan	7.28-7.30
UDP-glucose	5.60-5.62

S5 Table. Pathway analysis results for the comparison of the MRC5 and WM983B cell lines.

Pathway	Total	Hits	Raw p	FDR	Impact
Starch and sucrose metabolism	18	2	0.0132	0.1883	0.4304
Taurine and hypotaurine metabolism	8	1	0.0778	0.3660	0.4285
beta-Alanine metabolism	21	2	0.0178	0.1883	0.3992
Alanine, aspartate and glutamate metabolism	28	4	0.0001	0.0096	0.3373
Glutathione metabolism	28	3	0.0023	0.0624	0.2631
Pyruvate metabolism	23	2	0.0211	0.1888	0.1913
Arginine and proline metabolism	36	3	0.0049	0.0972	0.1883
Glycolysis or Gluconeogenesis	26	2	0.0268	0.2014	0.0979
Folate biosynthesis	27	1	0.2404	0.7636	0.0911
Arginine biosynthesis	14	3	0.0003	0.0113	0.0638
Pentose and glucuronate interconversions	19	1	0.1755	0.6687	0.0602
Amino sugar and nucleotide sugar metabolism	42	1	0.3494	0.9317	0.0575
Pantothenate and CoA biosynthesis	20	2	0.0162	0.1884	0.0476
Citrate cycle (TCA cycle)	20	1	0.1839	0.6688	0.0463
Galactose metabolism	27	2	0.0287	0.2014	0.0372
Purine metabolism	70	3	0.0302	0.2014	0.0145
Glycerophospholipid metabolism	36	1	0.3077	0.8791	0.0093
Primary bile acid biosynthesis	46	1	0.3759	0.9699	0.0075
Glyoxylate and dicarboxylate metabolism	32	2	0.0394	0.2383	0.0000
Glycine, serine and threonine metabolism	33	2	0.0417	0.2383	0.0000
Pyrimidine metabolism	39	2	0.0565	0.2944	0.0000
Nitrogen metabolism	6	1	0.0589	0.2944	0.0000
Ascorbate and aldarate metabolism	9	1	0.0871	0.3871	0.0000
Nicotinate and nicotinamide metabolism	15	1	0.1411	0.5944	0.0000
Histidine metabolism	16	1	0.1498	0.5995	0.0000
Propanoate metabolism	22	1	0.2004	0.6972	0.0000
One carbon pool by folate	26	1	0.2325	0.7636	0.0000
Lipoic acid metabolism	28	1	0.2481	0.7636	0.0000
Cysteine and methionine metabolism	33	1	0.2859	0.8471	0.0000
Tyrosine metabolism	42	1	0.3493	0.9316	0.0000

Total: Total number of compounds in the pathway. Hits: Matched number from the uploaded data. P-value: Original p-value calculated from the enrichment analysis. FDR: adjusted P-value using false discovery rate. Impact: Pathway impact value calculated from pathway topology analysis

S6 Table. Pathway analysis results for the comparison of the MRC5 and HT168M1 cell lines.

Pathway	Total	Hits	Raw p	FDR	Impact
Phenylalanine. tyrosine and tryptophan biosynthesis	4	1	0.0401	0.3561	0.5000
Starch and sucrose metabolism	18	2	0.0135	0.1539	0.4304
Phenylalanine metabolism	8	1	0.0786	0.5241	0.3571
Alanine. aspartate and glutamate metabolism	28	3	0.0024	0.0483	0.3373
Glutathione metabolism	28	3	0.0024	0.0483	0.2631
Arginine and proline metabolism	36	1	0.3105	1.0000	0.1640
Tryptophan metabolism	41	1	0.3456	1.0000	0.1431
Nicotinate and nicotinamide metabolism	15	2	0.0094	0.1255	0.1382
Inositol phosphate metabolism	30	1	0.2660	0.9671	0.1294
Arginine biosynthesis	14	3	0.0003	0.0234	0.0609
Pentose and glucuronate interconversions	19	1	0.1773	0.8173	0.0602
Amino sugar and nucleotide sugar metabolism	42	1	0.3525	1.0000	0.0576
Glycerophospholipid metabolism	36	2	0.0499	0.3989	0.0575
Galactose metabolism	27	3	0.0022	0.0483	0.0373
Purine metabolism	70	2	0.1567	0.8173	0.0071
Ascorbate and aldarate metabolism	9	2	0.0033	0.0535	0.0000
Neomycin. kanamycin and gentamicin biosynthesis	2	1	0.0202	0.2022	0.0000
Nitrogen metabolism	6	1	0.0595	0.4329	0.0000
Histidine metabolism	16	1	0.1514	0.8173	0.0000
Selenocompound metabolism	20	1	0.1857	0.8173	0.0000
Ether lipid metabolism	20	1	0.1857	0.8173	0.0000
Pantothenate and CoA biosynthesis	20	1	0.1857	0.8173	0.0000
beta-Alanine metabolism	21	1	0.1941	0.8173	0.0000
Pyruvate metabolism	23	1	0.2106	0.8425	0.0000
Glycolysis / Gluconeogenesis	26	1	0.2348	0.8945	0.0000
Glyoxylate and dicarboxylate metabolism	32	1	0.2811	0.9777	0.0000
Pyrimidine metabolism	39	1	0.3318	1.0000	0.0000

Total: Total number of compounds in the pathway. Hits: Matched number from the uploaded data. P-value: Original p-value calculated from the enrichment analysis. FDR: adjusted P-value using false discovery rate. Impact: Pathway impact value calculated from pathway topology analysis

S7 Table. Pathway analysis results for the comparison of the HT168M1 and WM983B cell lines.

Pathway	Total	Hits	Raw p	FDR	Impact
Taurine and hypotaurine metabolism	8	2	0.0026	0.0410	0.8286
beta-Alanine metabolism	21	2	0.0178	0.1582	0.3992
Alanine, aspartate and glutamate metabolism	28	5	4.43e-06	0.0004	0.3109
Glutathione metabolism	28	3	0.0023	0.0410	0.2827
Nicotinate and nicotinamide metabolism	15	1	0.1412	0.6644	0.2346
Histidine metabolism	16	2	0.0105	0.1047	0.2213
Glycine, serine and threonine metabolism	33	2	0.0417	0.3034	0.2146
Pyruvate metabolism	23	1	0.2086	0.6953	0.1914
Arginine biosynthesis	14	2	0.0080	0.0918	0.1223
Glycolysis or Gluconeogenesis	26	1	0.2326	0.7091	0.0978
Pentose and glucuronate interconversions	19	1	0.1755	0.6688	0.0602
Amino sugar and nucleotide sugar metabolism	42	1	0.3494	0.8470	0.0575
Pantothenate and CoA biosynthesis	20	1	0.1839	0.6688	0.0476
Citrate cycle (TCA cycle)	20	1	0.1839	0.6688	0.0463
Glyoxylate and dicarboxylate metabolism	32	4	0.0002	0.0083	0.0266
Cysteine and methionine metabolism	33	2	0.0417	0.3034	0.0218
One carbon pool by folate	26	1	0.2326	0.7091	0.0185
Starch and sucrose metabolism	18	1	0.1671	0.6688	0.0097
Primary bile acid biosynthesis	46	1	0.3759	0.8844	0.0076
Galactose metabolism	27	1	0.2404	0.7091	0.0022
Nitrogen metabolism	6	2	0.0014	0.0370	0.0000
Arginine and proline metabolism	36	3	0.0049	0.0648	0.0000
Pyrimidine metabolism	39	2	0.0565	0.3769	0.0000
Valine, leucine and isoleucine biosynthesis	8	1	0.0778	0.4787	0.0000
Ascorbate and aldarate metabolism	9	1	0.0871	0.4978	0.0000
D-Amino acid metabolism	15	1	0.1412	0.6644	0.0000
Butanoate metabolism	15	1	0.1412	0.6644	0.0000
Selenocompound metabolism	20	1	0.1839	0.6688	0.0000
Propanoate metabolism	22	1	0.2005	0.6953	0.0000
Lipoic acid metabolism	28	1	0.2482	0.7091	0.0000
Porphyrin metabolism	31	1	0.2710	0.7427	0.0000
Sphingolipid metabolism	32	1	0.2785	0.7427	0.0000
Valine, leucine and isoleucine degradation	40	1	0.3357	0.8470	0.0000
Tyrosine metabolism	42	1	0.3494	0.8470	0.0000
Purine metabolism	70	1	0.5147	1.0000	0.0000

Total: Total number of compounds in the pathway. Hits: Matched number from the uploaded data. P-value: Original p-value calculated from the enrichment analysis. FDR: adjusted P-value using false discovery rate. Impact: Pathway impact value calculated from pathway topology analysis
